# Analyzing the Functional Properties of the Creatine Kinase System with Multiscale ‘Sloppy’ Modeling

**DOI:** 10.1371/journal.pcbi.1002130

**Published:** 2011-08-11

**Authors:** Hannes Hettling, Johannes HGM van Beek

**Affiliations:** 1 Centre for Integrative Bioinformatics VU (IBIVU), VU University Amsterdam, Amsterdam, The Netherlands; 2 Section Medical Genomics, Department of Clinical Genetics, VU University Medical Centre, Amsterdam, The Netherlands; University of California Santa Barbara, United States Of America

## Abstract

In this study the function of the two isoforms of creatine kinase (CK; EC 2.7.3.2) in myocardium is investigated. The ‘phosphocreatine shuttle’ hypothesis states that mitochondrial and cytosolic CK plays a pivotal role in the transport of high-energy phosphate (HEP) groups from mitochondria to myofibrils in contracting muscle. Temporal buffering of changes in ATP and ADP is another potential role of CK. With a mathematical model, we analyzed energy transport and damping of high peaks of ATP hydrolysis during the cardiac cycle. The analysis was based on multiscale data measured at the level of isolated enzymes, isolated mitochondria and on dynamic response times of oxidative phosphorylation measured at the whole heart level. Using ‘sloppy modeling’ ensemble simulations, we derived confidence intervals for predictions of the contributions by phosphocreatine (PCr) and ATP to the transfer of HEP from mitochondria to sites of ATP hydrolysis. Our calculations indicate that only 15±8% (mean±SD) of transcytosolic energy transport is carried by PCr, contradicting the PCr shuttle hypothesis. We also predicted temporal buffering capabilities of the CK isoforms protecting against high peaks of ATP hydrolysis (3750 µM*s^−1^) in myofibrils. CK inhibition by 98% *in silico* leads to an increase in amplitude of mitochondrial ATP synthesis pulsation from 215±23 to 566±31 µM*s^−1^, while amplitudes of oscillations in cytosolic ADP concentration double from 77±11 to 146±1 µM. Our findings indicate that CK acts as a large bandwidth high-capacity temporal energy buffer maintaining cellular ATP homeostasis and reducing oscillations in mitochondrial metabolism. However, the contribution of CK to the transport of high-energy phosphate groups appears limited. Mitochondrial CK activity lowers cytosolic inorganic phosphate levels while cytosolic CK has the opposite effect.

## Author Summary

Creatine kinase (CK) has several functions in cellular energy metabolism. It catalyzes the reversible transfer of high-energy phosphate from ATP to creatine, facilitating storage of energy in the form of phosphocreatine. In muscle cells, this extra energy buffer plays a pivotal role in maintaining ATP homeostasis. Another proposed function of CK is the transport of energy from ATP producing to ATP consuming sites via a shuttle mechanism involving a mitochondrial and a myofibrillar isoform of CK. The extent to which this ‘phosphocreatine shuttle’ mechanism is used in muscle and other tissues is hotly debated. We use a computational model of the CK system which can predict energy transport and buffering of high demand peaks to estimate the relative importance of both roles in heart muscle. We validate the model with multiscale data on the level of enzyme kinetic constants and with dynamic oxygen consumption measurements in rabbit hearts. Since model predictions can be strongly affected by changes in parameter values, we employ ‘sloppy’ ensemble modeling which allows to set confidence regions for predictions. Our results indicate that the main function of CK in heart muscle lies more in temporal energy buffering of high peaks in ATP consumption during cardiac contraction than in energy transportation.

## Introduction

It is well established that creatine kinase (CK) catalyzes the reversible transfer of phosphate from ATP to creatine (Cr):
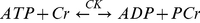
(1)


**Figure 1:**
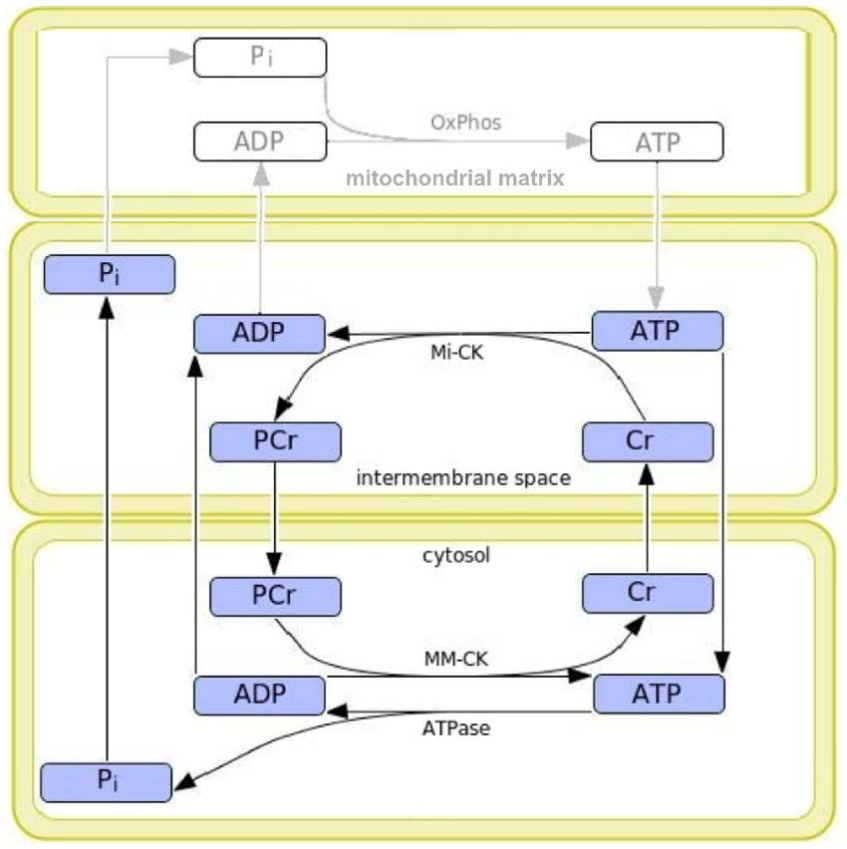
Scheme of model of the compartmentalized creatine kinase system. Main elements are ATP hydrolysis by ATPase, ATP synthesis by mitochondria and creatine kinase (CK) isoforms in the mitochondrial intermembrane space (Mi-CK) and cytosol (MM-CK). Oxidative phosphorylation (OxPhos) takes place in the mitochondrial matrix and responds to ADP and inorganic phosphate (P_i_) levels in the mitochondrial intermembrane space. The concentrations of phosphocreatine (PCr), creatine (Cr), ADP, ATP and P_i_ are dependent on the rates of the enzyme reactions and transport. The figure was generated with CellDesigner [57].

However, how this biochemical function plays a role in cell functioning has been the subject of intense controversy [1]. It is remarkable that two distinct isoforms of CK are expressed in muscle cells, one in the mitochondrial inner membrane space (IMS) and one in the cytosol where the contractile elements are located. This led to the idea of the ‘phosphocreatine shuttle’, proposed by Bessman and Geiger [2]: PCr formation from adenine nucleotide and creatine in the IMS is catalyzed by the mitochondrial isoform of CK, Mi-CK, located in the IMS. PCr may then proceed to the cytosol, which constitutes a mechanism of facilitated diffusion of high-energy phosphate (HEP) groups. Retransfer of HEP to adenine nucleotide to energize myofibrillar contraction is done by the muscular isoform of CK, MM-CK, located in the cytosol (see Figure 1). Transfer of HEP was argued to be accomplished either by direct diffusion of ATP through the mitochondrial outer membrane (MOM) and cytosol or indirectly via the ‘phosphocreatine shuttle’. The phosphocreatine shuttle hypothesis has led to extensive scientific debates on the role of CK, e.g. [1], [3], [4].

Besides the energy transfer function, the creatine kinase system was thought to be responsible for (i) temporal energy buffering by maintaining an adequate ATP/ADP ratio during interruption of energy supply [5] or during changing energy demand [3], [6] and (ii) for regulation of oxidative phosphorylation [7]. The CK system, transporting creatine instead of ADP from the cytosol to the mitochondria, is a potential key regulator of oxidative phosphorylation. CK inhibition experiments on rabbit hearts [8], [9] and CK knockout experiments in mice [10] suggest that the creatine kinase system affects the dynamic adaptation of oxidative phosphorylation to energy demand.

Mathematical modeling has proven helpful to understand the CK system: several existing models account for a compartmentalized energy metabolism system in myocytes under various conditions [6], [11]–[16]. The main differences between the model analyzed here and other models described in the literature are addressed in the Discussion. We build on a previously published computational model for the dynamic adaptation of oxidative phosphorylation to changing workloads which captures the key elements responsible for buffering and transport of HEP between IMS and cytosol [17], [18]. The model incorporates synthesis of ATP from ADP by oxidative phosphorylation in the mitochondria and ATP consumption in the cytosol, the reversible transfer of phosphate groups from ATP to creatine by CK enzyme reactions and metabolite diffusion between IMS and cytosol through the MOM (see Figure 1). The model's dynamic behavior is affected by 22 free parameters for enzyme kinetics and membrane permeability, which had been determined experimentally and were collected from the scientific literature.

In recent work we investigated the sensitivity of the predictions of this CK model with respect to possible error in the parameters using a simplified ensemble approach and found that even a modest error on each model parameter results in a broad range of possible predictions [19]. However, models containing many molecular kinetic parameters, all known with little accuracy, can yield useful predictions as long as the correlation of these inaccuracies is taken into account. Brown et al. showed, using a computational model of nerve growth factor signaling, that viable model predictions can be achieved in spite of a high degree of uncertainty in all kinetic parameters [20], [21]. The approach identifies so-called ‘sloppy’ combinations of parameters, which, when jointly altered, do not significantly change the outcome of a model simulation, meaning that multiple combinations of parameters describe experimental data equally well. Gutenkunst et al. investigated a variety of metabolic and signaling networks and found these spectra of correlated parameter sensitivities to be universal in Systems Biology models [22]. To use the information from these hidden correlations between parameters, a Bayesian ensemble of distinct parameter sets which agree with experimental data, can be sampled with Markov-Chain Monte Carlo (MCMC) methods. The likelihood of a parameter combination being included in the ensemble is proportional to the parameter combination's likelihood to predict the experimental input data set. Starting point for the walk through parameter space is the parameter set obtained from a least-squares parameter fit to experimental data. The resulting ensemble of parameter sets, constrained by the experimental data, allows a quantification of uncertainty not only of parameter values, but also delineates the uncertainty of model predictions for new experimental interventions. Below we demonstrate that combining molecular kinetic data, organellar data and whole organ response data with a sloppy modeling approach is feasible and fruitful.

We assembled a set of prior knowledge data on kinetic parameters of the CK enzymes and made use of measurements on the oxidative capacity and kinetics of isolated mitochondria and on metabolite transport across membranes and cytosol. These data at the molecular and organellar level were combined with experimental data on the response of the whole heart: for jumps to multiple heart rate levels the response time of the increase in oxygen uptake in the heart was measured. Based on model analysis of the oxygen transport system, the response time of oxygen uptake at the level of the mitochondria could be calculated from the whole heart level uptake [9]. These response times for wild type CK levels and during CK inhibition played an important role as input data for the MCMC analysis. Based on these data from multiple levels in the system, we predict the contribution of PCr to HEP transport and the buffering capacity of the system toward the high-frequency high-amplitude pulsations of ATP hydrolysis during the cardiac cycle. As a consequence, we determined that the functional role of the CK system in energy transport is limited and that high pulses in ATP hydrolysis are buffered by CK at order 100 millisecond time scales; both functions are presently not directly accessible to experimental measurement. Surprisingly, we also find that the mitochondrial CK isoform plays a role in regulating the cytosolic inorganic phosphate level.

## Results

We employed experimental data from three scales: molecular kinetic parameters, organellar capacity parameters and whole organ dynamic response data. A priori experimental information about kinetic parameters was extracted from the literature (see Table 1). For nine of the 22 model parameters, standard measurement errors were reported. In order to constrain these parameters by their measurement errors, we added this molecular and organellar information as terms to a least-squares cost-function which also contained dynamic response times measured at the whole heart level (see Methods). In this way experimental data from the molecular, organellar and whole system level are treated in a unified way. For the MOM permeability to adenine nucleotides (PS_mom,AdN_), a key parameter affecting the system's energy transport and buffering behavior, values in literature were contradictory [18]. The parameter PS_mom,AdN_ was therefore not constrained. The cost function determines the probability that a parameter set is compatible with the observed data (see Methods). Using Markov Chain Monte Carlo, a distribution of parameter sets with high probability of agreement with the data is sampled. The resulting ensemble of parameter sets is therefore a multivariate posterior distribution, shaped by the cost function, which reflects the probability of individual parameter sets in a Bayesian sense [21].

**Table 1:** Parameters of the CK model.

Name	Description	Value	Unit	Reference	Optimized value	Prior 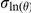	Ensemble mean±SD	Posterior 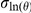
K_eq,CK_	Equilibrium constant for Mi-CK and MM-CK	152.0±4.0		[58]	151.95	0.026	152.32±3.82	0.025
*Parameters for the mitochondrial creatine kinase reaction*								
V_max,Mi,f_	Maximum velocity Mi-CK (PCr production)	882.0	µM/s	[18]	775.05	0.336^a^	760.05±264.39	0.333
K_ia,Mi_	Binary dissociation constant ATP	750.0±60.0	µM	[28]	751.32	0.081	754.79±62.61	0.083
K_ib,Mi_	Binary dissociation constant Cr	28800±8450	µM	[28]	28733.44	0.336	29742±10117	0.332
K_ic,Mi_	Binary dissociation constant ADP	204.0	µM	[11]	201.73	0.336^a^	221.03±79.15	0.337
K_id,Mi_	Binary dissociation constant PCr	1600.0±200.0	µM	[28]	1597.69	0.128	1597.04±190.54	0.118
K_b,Mi_	Ternary dissociation constant Cr	5200.0±300.0	µM	[28]	5209.08	0.058	5196.36±302.73	0.058
K_d,Mi_	Ternary dissociation constant PCr	500.0±20.0	µM	[11], [59]	499.51	0.040	502.19±20.64	0.041
*Parameters for the myofibrillar creatine kinase reaction*								
V_max,MM,f_	Maximum velocity MM-CK (ATP production)	11441.78	µM/s	[18]	7373.07	0.336^a^	7769.77±2591.30	0.308
K_ia,MM_	Binary dissociation constant ATP	900.0	µM	[11]	1026.24	0.336^a^	1033.59±351.91	0.336
K_ib,MM_	Binary dissociation constant Cr	34900	µM	[11]	34504.19	0.336^a^	36772±12695	0.330
K_ic,MM_	Binary dissociation constant ADP	222.4	µM	[11]	212.26	0.336^a^	225.49±78.53	0.338
K_id,MM_	Binary dissociation constant PCr	4730.0	µM	[11]	4516.55	0.336^a^	4955.05±1692.93	0.329
K_b,MM_	Ternary dissociation constant Cr	15500±2500	µM	[11], [59]	16744.44	0.167	16869±2940	0.177
K_d,MM_	Ternary dissociation constant PCr	1670±40	µM	[11], [59]	1669.76	0.024	1670.91±38.38	0.023
*Parameters for mitochondrial ATP production*								
V_max,syn_	Maximum ATP synthesis velocity	1503.74±152.65	µM/s	[60]	1332.64	0.103	1320.53±113.50	0.085
K_adp_	Apparent K_m_ mitochondria for ADP	25.0	µM	[18], [24]	35.88	0.336^a^	34.61±7.80	0.228
K_pi_	Apparent K_m_ mitochondria for P_i_	800.0	µM	[18], [24]	346.57	0.336^a^	378.88±118.91	0.296
*Permeabilities of the mitochondrial outer membrane*								
PS_mom,AdN_	Membrane conductance ATP and ADP	13.3	s^−1^	[18]	23.64	None	31.74±16.58	0.500
PS_mom,PCr_	Membrane conductance PCr	162.5	s^−1^	[18]	155.49	0.336^a^	167.42±57.39	0.334
PS_mom,Cr_	Membrane conductance Cr	162.5	s^−1^	[18]	154.20	0.336^a^	163.06±59.68	0.350
PS_mom,Pi_	Membrane conductance P_i_	194.0	s^−1^	[18]	195.63	0.336^a^	199.25±68.34	0.324

Shown are all model parameters describing the enzyme kinetics and transport across the mitochondrial outer membrane. The thirds column gives the parameter values obtained from the literature. If a standard measurement error could be obtained from literature, the value is given. We also give the parameter values after least-squares optimization to experiment data. We finally give means and standard deviations of the parameter ensemble. Note that the model parameters for maximum backwards velocity of both CK reactions, V_max,Mi,b_ and V_max,MM,b_ are not listed because their values are dynamically calculated from other parameter values via the Haldane relation: 
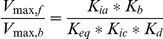

a
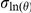
values which determine the spread of the prior distribution for parameters with standard errors not available from the experimental literature were set to 0.336, which is the maximum 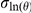 value for parameters where the standard error is known from the literature.

**Figure 2:**
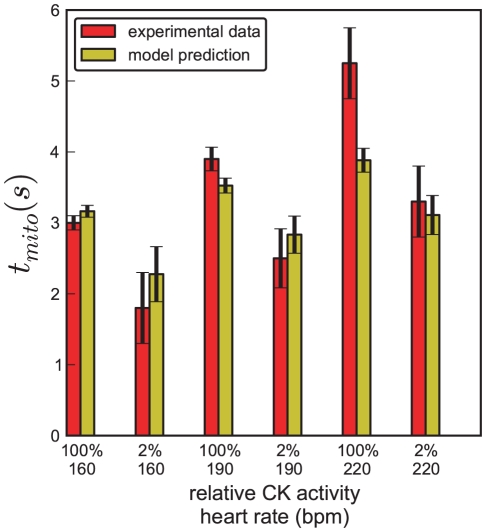
Fit by the model of measured response times to heart rate steps. The response times of oxidative phosphorylation (t_mito_) were measured in isolated rabbit hearts [9]. Model parameters were estimated using a modified Levenberg-Marquardt algorithm. Red bars represent the t_mito_ values from the experiment, yellow bars represent the t_mito_ values predicted by the model after the fitting procedure. Data is available for six different conditions: three different amplitudes of heart rate jump (from 135 bpm to 160, 190 and 220 bpm heart rate), each one measured with full wildtype CK activity (100%) or with CK activity inhibited to 2% of wildtype value. The error bars reflect the standard error of the measurements and the standard deviation of the t_mito_ values in the ensemble, respectively.

Data on the response times of the whole system level were taken from a study by Harrison et al., where electrically paced perfused rabbit hearts were exposed to a step increase in heart rate [9]. After applying the challenge, the metabolic delay time t_mito_ was calculated from dynamic O_2_ consumption measurements to estimate the generalized time constant of the ATP production time course. From a baseline level of 135 beats/min (bpm), heart rate was increased to 160, 190 and 220 bpm, respectively. Hearts were either exposed to iodoacetic acid (IAA) to block glycolysis or to iodacetamide (IA) to inhibit both glycolysis and CK activity, yielding in total 6 data points on the response time of oxidative phosphorylation, shown in Figure 2. Details on model, experimental data, cost function and the ensemble modeling approach can be found in the Methods section.

### Parameter estimation

Model parameters were estimated simultaneously to fit the t_mito_ values for all conditions using a least-squares optimization procedure. Different optimization algorithms (downhill simplex algorithm, Powell's method, Levenberg-Marquardt) gave similar quality of the fit. Initial and optimized parameter values can be found in Table 1. Figure 2 shows all t_mito_ values predicted by the model before and after parameter optimization for all conditions. After fitting, the model correctly predicts a quicker energy supply-demand signaling when CK is inhibited by 98%, causing weaker ADP/ATP buffering by CK. In the optimization procedure, the maximum velocities of the Mi-CK and the MM-CK enzyme were decreased by 12 and 36%, respectively, from their initial literature values. These literature enzyme activities for MM-CK and Mi-CK had been taken from the same experimental model, but without inhibition of glycolysis by IAA [8]. The experimental data used in the present analysis was measured in the presence of IAA which was found to impede CK activity by 20% [9]. The drop in estimated CK activity is therefore plausible. Other parameters which are altered significantly by the optimization are the apparent Michaelis constant for inorganic phosphate in the mitochondrion, K_pi_, which drops from 800 to 347 µM, and the apparent K_M_ for ADP, K_adp_, which increases from 25 to 36 µM. Both parameters occur in the model equation determining the rate of oxidative phosphorylation, which may explain the inverse variation. There exist *in vitro* measurements of K_pi_ that are lower than the initial value used in this analysis [18]: Stoner & Sirak for instance measured K_pi_ to be 360 µM [23] which is close to our optimized value. Likewise, reported values for K_adp_ vary between 20 and 30 µM [24], [25], corroborating the values obtained by the fit.

### Monte Carlo sampling of parameter sets

**Figure 3:**
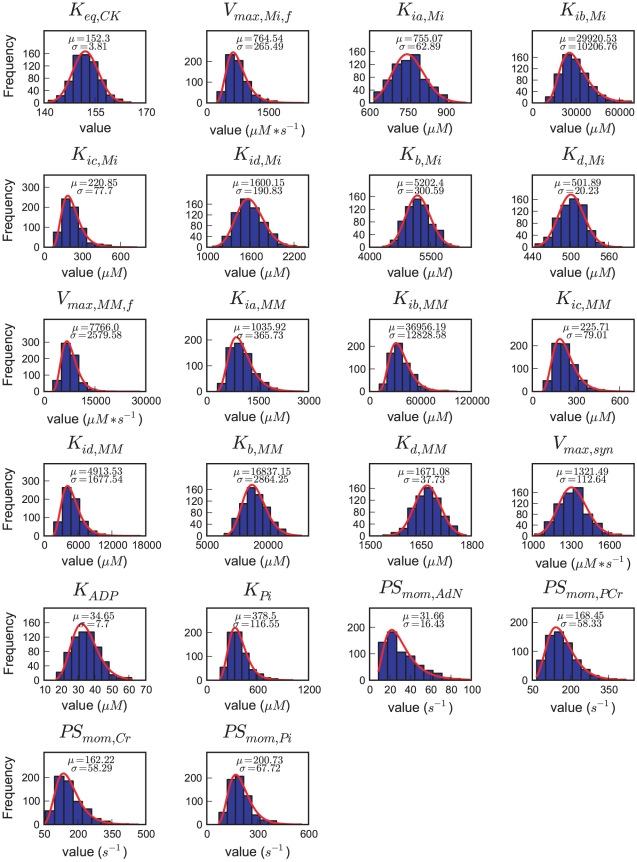
Distributions of individual parameters in the ensemble generated by the Metropolis-Hastings algorithm. Plots show histograms of all values in the ensemble for the given parameter. The ensemble consists of 658 parameter sets. Plotted in red is the probability density function of the lognormal distribution with mean and standard deviation of each parameter scaled to the observed frequencies.

Starting from the optimized parameter set (see Table 1), we sampled the parameter space to generate an ensemble of 658 independent parameter sets using the Metropolis-Hastings algorithm. The parameter set yielding the lowest cost in the complete ensemble was this optimized parameter set. The distributions of all parameters in the ensemble are shown in Figure 3. The nine kinetic parameters which had known error values (see Table 1) show a mean value in the ensemble close to the measured value and a standard deviation close to their reported measurement error from the literature, which was to be expected given the prior information in the cost function. However, the parameters for which there was no standard error value available from the literature in general gave a standard deviation in the ensemble which was smaller than the default assigned large standard error (see Table 1). We tested the effect of different assumptions on the default prior standard deviations on posterior parameter distributions and ensemble predictions, reported in Text S1 which shows that the conclusions reported here are not changed by larger or smaller values on the default prior.

The mean value of PS_mom,AdN_ in the ensemble is 31.7 s^−1^, which is larger than the optimized value of 13.3 s^−1^ found previously [18]. The distribution of PS_mom,AdN_ shows substantial skewing with a minimum value of 7.4 s^−1^, and a rather sharp exclusion of small values which give slow response times of the system. Based on experiments in isolated permeabilized cardiomyocytes, Sepp et al. ([26]) estimated a value for MOM permeability to adenine nucleotides of 1833 nmol/min/mg protein per mM concentration difference. Converting this value expressed per mg tissue protein, assuming 150 mg protein per gram wet weight, this corresponds to PS_mom,AdN_  =  7.45±1.89 s^−1^. This is virtually the same as the minimum estimated in our ensemble analysis.

### Predicting the contribution of PCr and ATP to energy transport

The contribution of PCr to intracellular HEP transfer, R_diff,PCr_, is quantified by the ratio of PCr diffusion (J_diff,PCr_) to the total phosphate group diffusion through the MOM:
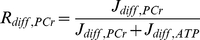
(2)


**Figure 4:**
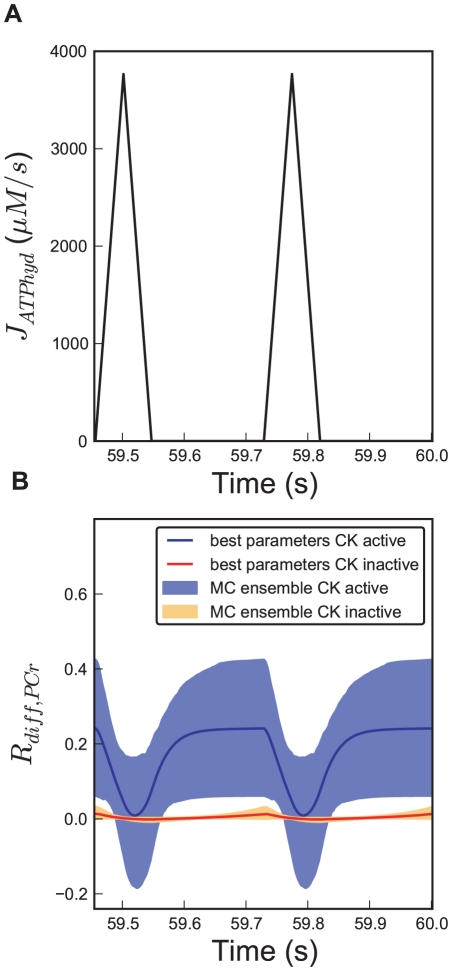
Prediction of energy transport from mitochondria to cytosol by PCr. (A) Forcing function of pulsatile cytosolic ATP hydrolysis for the last two cardiac cycles of a simulation over 60 s. (B) Prediction of the relative PCr contribution to high-energy phosphate flux across the mitochondrial outer membrane (R_diff,PCr_) at heart rate 220 bpm. The shaded region gives the central 95% confidence interval of the R_diff,PCr_ trajectories derived from ensemble simulations of 658 parameter sets. Solid lines depict a single simulation of the best scoring parameter set. Blue color indicates the condition with CK active. Simulations with CK inhibited by 98% by IA are plotted in orange. Note that two cardiac cycles are plotted after a steady state was reached.

An ensemble of simulations based on the parameter ensemble described above allows evaluation of the confidence region for the model prediction. In the ensemble, R_diff,PCr_ is on average 0.17±0.09 (mean±SD) at heart rate 160 bpm and 0.15±0.08 at 220 bpm in the case of active CK. Figure 4 shows the 95% confidence interval between upper and lower bound of the ensemble prediction for R_diff,PCr_ for IAA and IA conditions in steady state at heart rate 220 bpm. The small oscillations during CK inhibition are due to the 2% residual activity of CK. The upper bound of the 95% confidence interval remains below 0.44 during the cardiac cycle for all simulated conditions.

**Figure 5 pcbi-1002130-g005:**
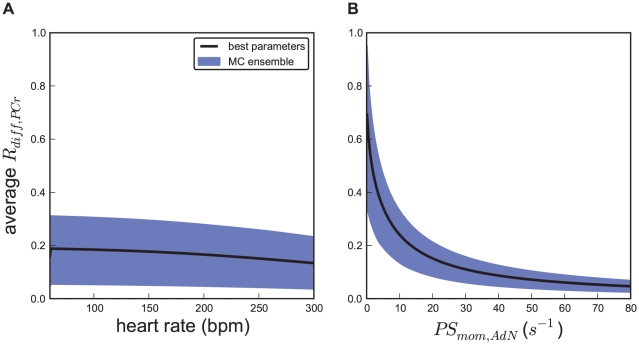
Dependence of PCr diffusion flux on heart rate and mitochondrial membrane permeability to adenine nucleotides. Prediction of the PCr contribution to high-energy phosphate flux across the mitochondrial outer membrane (R_diff,PCr_), averaged over the cardiac cycle, as a function of (A) heart rate and (B) mitochondrial outer membrane permeability for adenine nucleotides (PS_mom,AdN_), respectively. Values for (A) Steady state values for R_diff,PCr_ as a function of heart rate (B) Steady state values for R_diff,PCr_ as a function of PS_mom,AdN_ at fixed heart rate of 220 bpm. We performed simulations for the ensemble of Figure 3, with the heart rate or PS_mom,AdN_ set according to the x-axis. Blue shaded regions depict the 95% confidence interval of the prediction, black solid lines show the prediction for the optimized parameters (see Table 1).

R_diff,PCr_ decreases during the peaks in ATP hydrolysis and even becomes negative for the lowest trajectories in the ensemble, which indicates that PCr diffuses back to the mitochondria at the end of systole (Figure 4). The simulations show for these cases that ADP diffuses into the IMS during the peaks of ATP hydrolysis, stimulating a reversal of the mitochondrial CK reaction to produce ATP from PCr, exactly as happens in the cytosol. For these lowest trajectories in the ensemble the CK activity per unit volume of the intermembrane space is higher than the CK activity per unit volume of the cytosol, causing the PCr to go down more steeply in the intermembrane space. This causes the cytosolic PCr concentration to exceed the PCr concentration in the IMS, and a negative gradient forces PCr to diffuse back into the IMS. However, when averaged over the cardiac cycle, R_diff,PCr_ is always positive, indicating net flux of PCr from the mitochondria to the cytosol, and for the vast majority of the ensemble PCr diffusion flux never becomes negative during the entire cardiac cycle. Simulations suggested that the relative importance of the PCr shuttle becomes less with higher ATP hydrolysis at heart rates of 160, 190 and 220 bpm. We tested this hypothesis by predicting R_diff,PCr_ for a range of heart rates from 60 to 300 bpm. The ensemble simulations reveal that R_diff,PCr_ continuously drops for increasing heart rates for all sampled parameter combinations (see Figure 5A). The predicted decline in R_diff,PCr_ and increase in P_i_ concentration agrees with results of a recent study on perfused rat hearts [27]. Increased energy demand induces an increased ATP gradient between both compartments. At 160 bpm, the average difference between the ATP concentration in IMS and cytosol is 18.6 µmol*l^−1^, at 220 bpm it becomes 22.3 µmol*l^−1^ for the optimal parameter set. The increased ATP gradient across the MOM induces direct ATP transport instead of facilitated transport via PCr.

In order to demonstrate the dependence of shuttle utilization on the membrane conductance for adenine nucleotides, we predicted R_diff,PCr_ as a function of PS_mom,AdN_ for the ensemble. The predicted range shown in Figure 5B indicates that only for very small ATP permeability, PCr contribution becomes high. Even for the minimum value for PS_mom,AdN_ in the ensemble (7.35 s^−1^), the entire 95% confidence interval of R_diff,PCr_ remains below 0.5. Low MOM permeability to adenine nucleotides causes high-energy phosphate group transport via PCr, and that PS_mom,AdN_ is never lower than 7.35 s^−1^ therefore argues against a predominant phosphocreatine transport. Also when the value PS_mom,AdN_  =  7.45 s^−1^ estimated from Sepp et al. ([26]), see above, is incorporated as prior knowledge, the analysis still yields similar predictions of R_diff,PCr_, which stays with 95% confidence between 0.16 and 0.46 at heart rate 220 bpm.

It might be argued that the K_ia_ value of the mitochondrial CK should be set to 290 µM with oxidative phosphorylation active ([28]) to reflect functional coupling of CK to the adenine nucleotide translocator (ANT). Optimization based on this K_ia_ value gives as result that on average 18% of the high-energy phosphate flux at a heart rate of 220 beats/min is transported in the form of PCr, the rest as ATP. The parameter values for V_max,Mi,f_ calculated from rat heart mitochondria is 1609±113 µM/s in [28] and V_max,ATPsyn_ is 2960 µM/s which is about twice the value measured in the rabbit heart study analyzed here. When using the rat heart parameters combined with K_ia_ = 290 µM, the contribution of PCr to high-energy phosphate transport is estimated to be 25%. Further analysis of a model which incorporates a microcompartment which functionally couples the mitochondrial creatine kinase to the adenine nucleotide translocator ([6]) shows that it is difficult to explain the response time and molecular kinetic parameters simultaneously with this model. The results of this analysis can be found in Text S2. The conclusion that the contribution of PCr to high-energy phosphate transport is relatively modest appears to be robust, because the contribution was estimated to be 15–17% in the ensemble study with rabbit heart parameters, see above, and does not become substantially higher in analyses with other parameter sets.

### Prediction of temporal energy buffering

**Figure 6 pcbi-1002130-g006:**
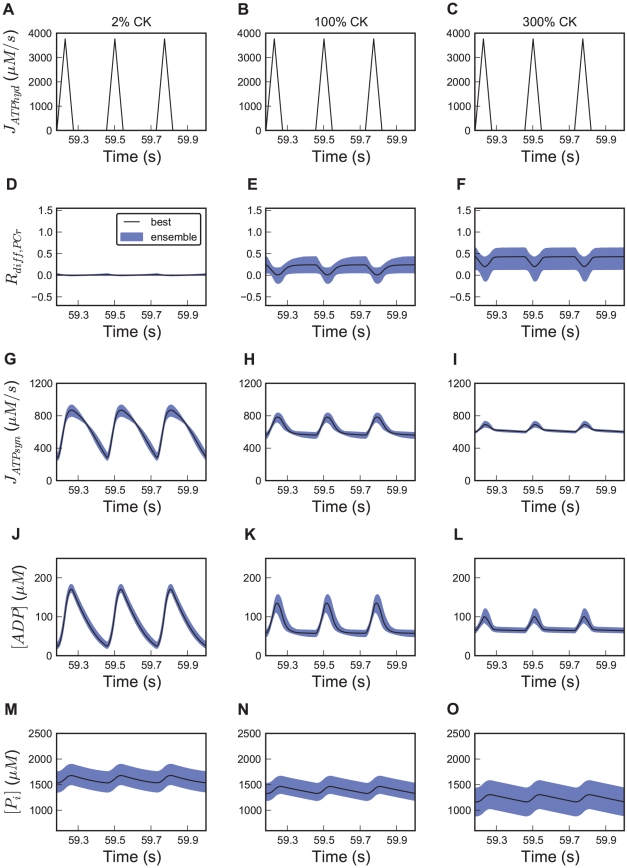
Fluctuations of metabolite concentrations and fluxes during the cardiac cycle at three levels of CK activity. Plots show (A–C) Trajectory of the forcing function of ATP hydrolysis and ensemble predictions of (D–F) R_diff,PCr_, (G–I) mitochondrial ATP synthesis rate, (J–L) cytosolic ADP and (M–O) cytosolic P_i_ concentrations at heart rate 220 bpm. Mi-CK and MM-CK activities were set to 2, 100, and 300% of wildtype levels. Three cardiac cycles are shown at steady state. Solid lines show the simulated trajectory of the optimized parameter set (see Table 1). Shaded regions show the 95% confidence interval for all trajectories of the ensemble of 658 parameter sets. To alter CK activity, the parameters describing maximum enzyme velocity, V_max,Mif_ and V_max,MMf_, are changed in parallel to the indicated percentage.

The results described above indicate that direct ATP transport is predominant in working heart muscle. Given that PCr energy shuttling is of limited importance, we investigated another potential function of CK, i.e. temporal energy buffering. When ATP consumption by the myofibrils exceeds mitochondrial ATP production during muscle contraction, ATP homeostasis can be maintained by PCr [4]. Ensemble predictions for R_diff,PCr_, concentrations of cytosolic ADP and P_i_ and ATP synthesis rate at relative CK activity of 2, 100, and 300% of wild type levels are shown in Figure 6. Note that Mi-CK and MM-CK activities are both changed by the same factor in this set of simulations. Even at 3-fold increased CK activity, R_diff,PCr_ does not increase dramatically (Figure 6F). However, oscillations of cytosolic ADP concentrations are significantly affected by the CK activity. The amplitude of the ADP oscillation is 77±11 µM at normal CK levels and becomes 146±1 µM if CK is inhibited by 98%, as is the case for IA treated perfused hearts (Figure 6K,J). At threefold increased CK activity this becomes 36±22 µM (Figure 6L). In simulations of a hypothetical case with 10000-fold increase of enzyme activity, oscillations of adenine nucleotide concentrations are almost fully damped to an amplitude of 2.6±0.2 µM (data not shown).

The time course of mitochondrial ATP production oscillates with amplitudes of 566±31, 215±23 and 91±14 µM*s^−1^ for 2, 100 and 300% relative CK activity, respectively (Figure 6G–I). The pulsatility of ATP and ADP concentrations and of ATP synthesis is synchronized to ATP hydrolysis in the myofibrils. The confidence regions for these trajectories are relatively narrow. By blocking CK by 98%, the average concentrations of ADP in the IMS increases to 64±9 µM from 56±9 µM at normal CK levels. In contrast to ADP, the amplitude of oscillations of cytosolic inorganic phosphate stays relatively constant at different CK activities at about 145 µM. This reflects that P_i_ is not directly buffered by CK. Surprisingly, average levels of cytosolic inorganic phosphate drop with CK activity. The average P_i_ concentration at 2% CK activity is 1618±97 µM and becomes 1416±80 µM for wild-type CK activity (Figure 6M,N). For all parameter sets in the ensemble the P_i_ concentration declines when CK activity is increased.

### The specific role of the mitochondrial CK isoform

Transport of HEP by PCr from mitochondria to cytosol partially takes place via the circuit formed by both CK isoforms, but was predicted to be quantitatively not very important. On the other hand, temporal buffering of the systolic ATP hydrolysis burst needs only the MM-CK activity in the cytosol, which is much higher than the Mi-CK activity (see Table 1). It was therefore still unclear what the function of the mitochondrial CK isoform is.

**Figure 7:**
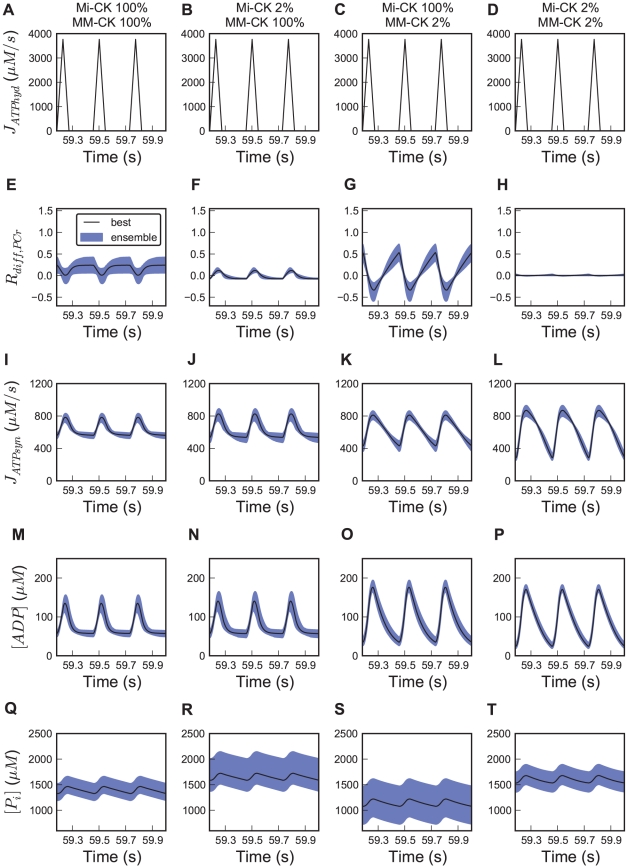
Ensemble predictions of metabolite concentration and flux oscillations during the cardiac cycle for selective CK isoform inhibition. In the first row (panels A–D), the pulsatile forcing function for ATP hydrolysis is plotted. Predictions of the time courses of (E–H) relative contribution of PCr to high-energy phosphate transport, R_diff,PCr_, (I–L) ATP synthesis rate, (M–P) cytosolic ADP and (Q–T) P_i_ concentrations. Heart rate is 220 bpm. In the four columns we compare: no CK inhibition, 98% Mi-CK inhibition, 98% MM-CK, or both CK enzyme reactions inhibited by 98%. Black solid lines show the simulated trajectory of the optimized parameter set (Table 1). Blue shaded regions show the 95% central confidence interval for all trajectories of the ensemble of 658 parameter sets. To alter CK activity, the parameters describing maximum enzyme velocity, V_max,Mif_ and V_max,MMf_, are changed to the indicated percentage. Three cardiac cycles are shown after a steady state was reached. Note that the first and the last column also appear in Figure 6 and are shown here for ease of comparison.

In order to further elucidate the effect of the compartmentalized CK system on metabolism, we performed ensemble predictions with individual inhibition of both CK isoforms one by one. In Figure 7, we show the 95% confidence intervals of predicted metabolite concentrations and reaction fluxes. The amplitude of oscillations in mitochondrial ATP synthesis is predicted to rise from 215±23 µM*s^−1^ at baseline CK activity to 278±33 with 98% Mi-CK inhibition, compared to 375±21 µM when MM-CK is inhibited by 98% (Figure 7I–K). Thus, despite its low activity, Mi-CK still has a small but clear effect on the ATP synthesis oscillation amplitude. Inhibition of Mi-CK has a larger effect when MM-CK is already inhibited (amplitude 565±31 µM*s^−1^, Figure 7L). The damping of ADP oscillation is highly affected by MM-CK but not by Mi-CK: 98% inhibition of Mi-CK leads to an increase in the amplitude of systolic ADP oscilation from 77±11 to 83±11 µM (Figure 7M,N), whereas MM-CK inhibition doubles the amplitude to 146±1 µM (Figure 7O).

Predictions of R_diff,PCr_ illustrate that both Mi-CK and MM-CK are required for a functioning phosphocreatine shuttle. PCr diffusion averaged over the cardiac cycle makes a very small contribution to total HEP delivered from the mitochondria when either Mi-CK or MM-CK is inhibited by 98%. With 98% inhibited Mi-CK activity, R_diff,PCr_ is even slightly below zero during diastole with low ATP hydrolysis, meaning that PCr is transported from cytosol to IMS (Figure 7F). Note that this situation is reversed with respect to normal Mi-CK and MM-CK activity where PCr diffusion is always positive during diastole and occasionally becomes negative during ATP hydrolysis peaks. For normal CK activity the explanation for reversed PCr diffusion during ATP hydrolysis (Figure 7E) was that the CK activity per unit volume is higher in the IMS than in the cytosol. During Mi-CK inhibition this is of course no longer the case and systolic PCr consumption in the cytosol leads to PCr diffusion from the IMS, explaining the reversal of PCr transport during systole. In contrast, with MM-CK inhibited, ATP is buffered by Mi-CK in the IMS and PCr diffuses to the IMS at the end of the ATP hydrolysis peaks. This explains why R_diff,PCr_ goes more negative during ATP hydrolysis peaks with MM-CK inhibition and its oscillation is stronger than for normal Mi-CK and MM-CK activity (Figure 7E, G). When inhibiting Mi-CK activity, our model predicts an increase in the amplitude of [ADP] oscillation in the IMS from 57±8 to 71±8 µM. Mi-CK therefore has a damping effect on oscillations of ADP concentrations in the IMS, which contributes to the damping of mitochondrial ATP synthesis.

The concentration of cytosolic P_i_ is predicted to be lowered by mitochondrial creatine kinase activity. Blocking Mi-CK leads to a P_i_ increase by about 18% from 1416±80 to 1670±167 µM (Figure 7Q, R). If Mi-CK is inhibited by 100%, the steady state P_i_ concentration becomes 1678±173 µM (data not shown). MM-CK inhibition decreases the P_i_ concentration; a combination of Mi-CK and MM-CK inhibition leads to a slightly higher P_i_ level compared to the wildtype (Figure 7S, T).

## Discussion

The relative importance of the different roles of the CK system in myocytes is still hotly debated [4]. The present study was designed to investigate the function of CK in cardiomyocytes under varying workloads. In particular we sought to elucidate whether the phosphocreatine shuttle is the major pathway for HEP transfer from mitochondria to energy consuming myofibrils as stated in the phosphocreatine shuttle hypothesis or whether CK has other metabolic functions, e.g. the damping of swings in ATP and ADP concentrations and oxidative phosphorylation.

Various computational studies of cardiac energy metabolism have been published based on models which contained the creatine kinase reaction, ATP hydrolysis and synthesis. The model analyzed in the present study is a subset of the model of Vendelin et al. ([6]) and was described previously [17], [18]. The diffusion gradients in the cytosol which had been shown to be very small ([6]) were replaced by a simple diffusion conductance. The adenine nucleotide translocator and phosphate carrier in the mitochondrial inner membrane and oxidative phosphorylation (OxPhos) reactions in the mitochondria in the model of Vendelin et al. were replaced by a Michaelis-Menten equation describing OxPhos flux as a function of ADP and Pi in the intermembrane space [18]. The model was further modified in order to prevent thermodynamically infeasible loops by introducing constraints on the equilibrium of the CK reactions in IMS and cytosol [19]. Some models in the literature implement substrate channeling between ANT and Mi-CK by a microcompartment which is located inside the intermembrane space [6], [11], [29]. The performance of those models is discussed below. Other models exist for myocardial energy metabolism which do not consider the role of two creatine kinase isoforms connected via facilitated diffusion. For instance, Beard et al. integrated a detailed model of oxidative phosphorylation [14] with a model of spatially distributed oxygen transport and HEP metabolism to investigate the regulation of oxidative phosphorylation at different cardiac workloads [5] and HEP buffering in hearts at high workloads, acute ischemia and reactive hyperemic recovery.

In the present study we predicted the functions of the CK enzyme isoforms based on the following strategy. A set of experimental data from multiple scales was assembled. We based the analysis on our model which had been shown to contain the key elements of the CK system [17], [18]. The experimental data set allowed to estimate all parameters of this model. In order to set confidence regions for the predictions of CK function, the experimental errors for the data were taken explicitly into account. This was possible by generating an ensemble of model parameter sets. The probability of a set of parameters being part of the ensemble was determined based on the probability of the predicted experimental data set given the parameters. This approach was termed sloppy modeling [21]. Brown et al. [20] and Gutenkunst et al. [22] applied it to time series of protein activity levels measured during dynamic responses of a system as a whole. The surprising finding in their studies was that responses of the system as a whole were predictable with acceptable confidence regions even if all parameters of the model were known with very poor accuracy. This is possible because the correlation between parameters is well defined by the behavior of the system as a whole.

A novel aspect in the present study is that we combined data taken from different integration levels in the system: kinetic parameters determined on enzymes in isolation, enzyme activity levels measured in tissue homogenates, organellar capacity levels measured on isolated mitochondria and dynamic response times determined on the heart as a whole. The whole organ response times were very important because they sensitively depend on the permeability of adenine nucleotides through the outer mitochondrial membrane, one of the organellar level parameters. This MOM permeability could not be determined experimentally with any degree of accuracy in isolated mitochondria. Combining some strategically important data from the whole system level with molecular parameters appears sufficient to predict system properties with acceptable confidence regions (Figures 4–7).

Many of the experiments that are invoked to support high degrees of functional coupling between CK and ANT have been done in isolated mitochondria or in isolated myocytes and muscle fibres that were permeabilized. These were often studied at temperatures substantially below the physiological level. An important aspect of our analysis is that we try to estimate the functional roles of CK in the intact heart. To that end we combine the kinetic data from the molecular level with data obtained in isolated perfused hearts. It is important to realize that these hearts were intact, with contractility and cell membranes fully functional. Our model analysis explains the experimental data without invoking direct coupling of CK and ANT. However, the limited permeability of the mitochondrial outer membrane to adenine nucleotides, estimated from the response time in the intact heart, results in a certain degree of dynamic compartmentation of the adenine nucleotides. This approach helps to define the functional roles of CK in the intact heart at physiological temperatures. If CK-ANT direct coupling is the only way that ADP is delivered to the ANT, then the experiments with 98% inhibition of CK cannot be explained. It would then also be hard to explain that Mi-CK knockout animals still have substantial cardiac contractile function [30]. Future CK-ANT interaction models need to address such experimental data sets with CK inhibition and also explain the phosphate-labeling data of Erickson-Viitanen et al. [31]

Our findings suggest that the principal role of the CK system in heart muscle is to serve as a temporal energy buffer for ATP and ADP at the 100 millisecond time scale. CK's role in supporting transport of high energy phosphate groups seems of limited importance. If oxygen supply is interrupted, PCr will also buffer ATP and ADP for several seconds [5]. Temporal energy buffering therefore has a relatively large bandwidth. Joubert et al. experimentally investigated the role of the CK shuttle by ^31^P NMR magnetization transfer protocols in vivo and proposed the hypothesis of a versatile role of PCr on intracellular energy transport depending on cardiac activity [32], [33]. Partial inhibition of ATP synthesis led to a decrease of indirect energy transport via PCr. This decrease is predicted by our model (data not shown). Some computational models on compartmentalized energy transfer in muscle, as for instance in Vendelin et al. ([13]), assume restricted diffusion of adenosine nucleotides to an extent where energy transport via PCr becomes essential. However, a large restriction of adenine nucleotide permeation of the cytosol and MOM is not compatible with the relatively fast responses of oxidative phosphorylation to cytosolic workload steps [18].

The conductance parameter PS_mom,AdN_ in our model reflects not only the permeation of the MOM proper but in series with that also diffusion in myofibrils and cytosol. The inverse of PS_mom,AdN_ in our model is therefore the sum of the inverse of permeability-surface products (PS) for the MOM proper and cytosol, respectively [18]. The present Monte-Carlo ensemble approach indicates that PS_mom,AdN_ lies within a range from 7.4 to 115 s^−1^ (see Figure 3). Based on the transverse diffusion coefficient of 52 µm^2^/s for ATP in the myofibrillar space measured with fluorescently labeled ATP [34], the PS calculated for the cytosol is 216.7 sec^−1^ [18]. Given an ensemble mean PS_mom,AdN_ of 31.7 s^−1^ (see Table 1) we predict that about 15% of the total resistance to diffusion can be attributed to the cytosol. Note that the fluorescently labeled ATP has a higher molecular mass than ATP. The true diffusion coefficient of ATP is probably higher and the contribution of the cytosolic space to diffusional resistance is therefore probably overestimated in this calculation. The contribution of PCr to HEP transport predicted in the present study (Figure 4) is compatible with measured response times of the system (Figure 2). It has been suggested that in cardiomyocytes the density of mitochondria and their vicinity to myofibrils is sufficient to ensure energy transport via adenosine nucleotides [3]. The prediction by our model that CK-facilitated transport of PCr is not obligatory for HEP transport is in line with the observation that CK knockout has relatively mild effects on cardiac function [10], [30], [35].

Activation of oxidative phosphorylation has been proposed to be strongly dependent on substrate channeling of ATP and ADP between the tightly coupled enzymes Mi-CK and ANT, meaning that ATP exported from the mitochondrial matrix via ANT is immediately available as a substrate for Mi-CK. The resulting ADP is then channeled back to stimulate oxidative phosphorylation in the mitochondrial matrix. However, the hypothesis of functional coupling is still debated [1] and other studies seem to contradict it [36]. In order to investigate the effect of functional coupling between the ANT and Mi-CK we implemented and analyzed the model of Vendelin et al. ([6]), where the reactions are coupled by a microcompartment (see Text S2). The model, which contains constants which phenomenologically reflect the functional coupling of Mi-CK to the ANT is considered to give a good and computationally effective representation of the functional coupling between Mi-CK and oxidative phosphorylation [29]. It appeared to be rather difficult to fit the model of Vendelin et al. to the given experimental data of mitochondrial delay times (t_mito_) when measurements on molecular kinetic parameters are taken into account in the cost function. Especially at low workloads, a quicker response to a step in ATP consumption rate after CK inhibition could not be predicted with this model. Even when all parameters from the model of Vendelin et al. were variable during the optimization procedure, the quality of the fit is far from optimal despite the fact that the model of Vendelin et al. has about three times as many parameters as our present model. We therefore do not consider the microcompartment explicitly in our present study.

The present results suggest that most of the delay of the activation of oxidative phosphorylation after temporal changes in ATP hydrolysis is caused by the delay of changes in phosphate metabolite levels outside the inner mitochondrial membrane. To investigate whether processes inside the mitochondria delay the response further, we tested a model of the mitochondrial matrix including metabolite transport across the inner mitochondrial membrane with instananeous step changes in ADP or P_i_ and also with ADP and P_i_ simultaneously outside the inner mitochondrial membrane. This corresponds to the model applied in Text S2 with all processes outside the inner mitochondrial membrane removed and the ADP and P_i_ concentrations outside the inner mitochondrial membrane set as forcing function. After a 20% increase in ADP concentration, ATP synthesis in the mitochondria reached a steady higher level within one second. The response time, calculated as for t_mito_, was 0.4 s. For a step in P_i_ the response was even faster with a negative value for the response time of −0.3 s because the response showed an overshoot. For a simultaneous change in ADP and P_i_ the mitochondrial response was essentially complete within half a second, with a response time of 0.08 s. When extramitochondrial ADP is changing, both mitochondrial oxygen consumption and ATP efflux via the ANT reacted even faster than the ATP synthase reaction. The fast response of mitochondrial metabolism predicted by the model is in agreement with spectroscopic measurements of the oxidation state of the electron carrier cytochrome b which was oxidized with a half-time of 70 milliseconds after a step in extramitochondrial ADP concentration at 26°C, and presumably much faster at the physiological temperature [37].

In studies on isolated rabbit cardiac muscle mitochondria the direct contribution of mitochondrial ATP to PCr formation by Mi-CK is low [31]. It was shown with radioactively labeled phosphate groups that if the concentration of ATP in the environment of the mitochondria is larger than 0.2 mM, less than 6% of PCr synthesis uses ATP synthesized immediately beforehand in the mitochondrial matrix. This is incompatible with a model where a major part of PCr is synthesized from ATP directly transferred to creatine kinase via a very small compartment with limited exchange with its environment.

By *in silico* analysis, we inferred distinct roles for the mitochondrial and myofibrillar creatine kinase enzymes. MM-CK is mainly responsible for damping large swings in metabolite concentrations and large oscillations in the rate of oxidative phosphorylation which would otherwise be caused by the large peaks of ATP hydrolysis during the cardiac cycle. Mi-CK restricts high concentrations of inorganic phosphate, which is surprising considering that inorganic phosphate is not handled directly by CK. Despite its low activity, Mi-CK also decreases oscillations of ATP synthesis, mainly due to the effect of Mi-CK on ADP oscillations in the intermembrane space.

The effect of the CK isoforms on the buffering of ADP oscillations and the prevention of high concentrations of inorganic phosphate may play a role in the prevention of formation of reactive oxygen species (ROS). ROS production highly depends on the mitochondrial membrane potential, which is increased at low ADP levels [38], [39]. The electric membrane potential in mitochondria can also be altered by inorganic phosphate, leading to enhanced ROS release [40]. Low ADP concentrations during diastole are prevented by MM-CK according to our predictions (see Figure 7). A protective role of Mi-CK against oxygen radical formation by preventing high inorganic phosphate concentrations is also predicted by our model. A function of Mi-CK to prevent oxygen radical formation has been found experimentally in isolated brain mitochondria [38]. The energy buffering role of the CK system has been linked to the prevention of oxidative stress in neurons [41], [42]. Creatine supplements to nutrition have also been shown to have a neuroprotective effect in models of Huntington's disease [43], [44]. The effects of creatine as a nutritional supplement in health and disease have recently been reviewed by Wallimann et al. [45].

In conclusion, we showed that by using a relatively small ‘skeleton’ model we were able to explain the dynamic adaptation of cardiac energy metabolism to changing workloads and to discern different functions of distinct CK isoenzymes. The sloppy modeling approach enables to make useful predictions of CK system behavior despite limited experimental input data and limited knowledge of kinetic parameters. The concept of sloppy modeling can also be used to find optimal experimental designs to further test the model [46]. We also demonstrated that combining a computational model analysis with experimental data on the level of cellular organelles and isolated enzymes and with the response of the heart as a whole provides a powerful combination that gives valuable insights in the functional roles of CK, such as regulation of oxidative phosphorylation, energy transport, inorganic phosphate levels and buffering of peaks of ATP hydrolysis at the 100 millisecond time scale.

## Methods

### Computational model

For our analysis, we employed a previously published computational model [18]. It is available in various formats and can be found in the BioModels database [47] as well as in the CellML model repository [48]. The model incorporates the key elements of the CK system with ATP synthesis in the mitochondria and pulsatile ATP hydrolysis in the cytosol (see Figure 1). The input of the model is a forcing function of cytosolic ATP usage catalyzed by myosin-ATPase and ion pumps. The model contains ten ordinary differential equations (ODEs) describing the rate of change of each metabolite concentration (ADP, ATP, PCr, Cr, P_i_) in two compartments over time. These equations were extensively described previously [18]. Model dynamics depend on 22 kinetic parameters retrieved from the literature which are listed in Table 1. In general the kinetic constants retrieved from the literature have relatively modest standard errors. However, for the permeability of the MOM to ATP and ADP (assumed to be equal in the model analysis; cf. [6]), reported values differed from 0.16 [6] to 85 µm*s^−1^ in the model of Beard [14] based on measurements of Lee et al. [49]. This large variation is possibly due to mitochondrial isolation or cell membrane permeabilization procedures.

The mitochondrial outer membrane permeability-surface product parameter PS_mom,AdN_ influences the response time for dynamic adaptation of oxidative phosphorylation strongly. Therefore the dynamic measurements of venous oxygen outflow in the heart as a whole in response to an increase of heart rate allow estimating the mitochondrial membrane permeability at the organellar level. The whole heart measurements were corrected for oxygen transport delay to reflect events at the level of the mitochondria (see below). The mitochondrial response time t_mito_ is defined as the generalized time constant of the time-course of oxygen consumption (defined to be equivalent to the first central statistical moment of the impulse response function in case the system is linear), previously described in [18], [50]-[52]. From a model simulation, t_mito_ is calculated as follows:
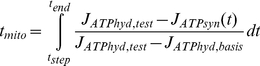
(3)


Where J_ATPhyd,basis_ and J_ATPhyd,test_ are the values for the ATP hydrolysis rates for the two electrically paced heart rates at baseline and test level, averaged over the cardiac cycle; J_ATPsyn_ denotes the time course of ATP synthesis in the mitochondrion. t_step_ is the time point when the heart rate is increased and t_end_ is the time point of the final oxygen measurement. Note that the average J_ATPsyn_ in the steady state before and at the end of a test challenge equals J_ATPhyd,basis_ and J_ATPhyd,test_, respectively.

**Figure 8:**
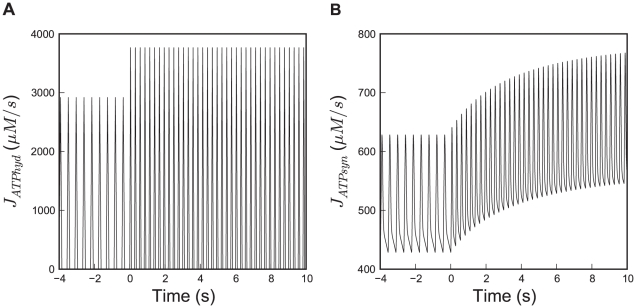
Pulsatile nature of energy production and consumption in the beating heart and the response to a step in heart rate. Shown are the time courses of (A) ATP hydrolysis and (B) synthesis simulated with the model of Figure 1. At time 0 s, average ATP hydrolysis rate was increased from 486.5 to 627.6 µM*s^−1^ corresponding to an increase in heart rate from 135 to 220 bpm, as was imposed in the experiments which were simulated in this study. Please note the difference in scale of the y-axis between panels A and B.

In order to correspond with the experimental conditions in [9], t_end_ was set to 60 seconds with t_step_ = 0 seconds; an initial run for 40 seconds before the heart rate step ensures that ATP synthesis has adapted to ATP hydrolysis and is found to be in steady state at this stage. In order to investigate the damping capabilities of the modeled system, ATP hydrolysis is simulated as a pulsatile function representing the alternating nature of energy demand in systole and diastole as described in [18]. Figure 8 shows the dynamic response of mitochondrial ATP production in response to a step in heart rate and ATP hydrolysis.

### Sloppy ensemble modeling

Almost all models in systems biology contain parameters that cannot be determined precisely. It is common practice to estimate missing parameter values by a parameter fit to experimental data. After the fit, one can make model predictions and analyze the underlying biological processes. This, however, is dangerous because a range of parameter combinations may agree with the available data equally well, potentially leading to deviating model predictions of new experimental situations. Directions in parameter space where parameter changes do change the simulation outcome very little were termed ‘sloppy’ by Brown et al., whereas directions where small changes in parameter values affect the dynamic behavior of the system strongly were termed ‘stiff’ [21]. Sloppy parameter sensitivity spectra have been identified for numerous biological models by the analysis of the eigenvectors and eigenvalues of a sensitivity matrix calculated from the chi-square cost function [22]. Sloppy models exhibit a characteristic pattern with the logarithms of eigenvalues approximately uniformly distributed over a large range. A sensitivity analysis of the CK model revealed the presence of both stiff and sloppy parameter combinations and a ‘sloppy’ sensitivity spectrum [53]. Since our model shows sloppy parameter sensitivities and is based on data subject to experimental variation, drawing predictions from an ensemble of parameter sets is preferable to merely relying on one parameter set fit to experimental data. According to the sloppy modeling paradigm ([21], [22]), the probability of a set of model parameters 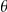 to be included in the ensemble is proportional to its likelihood to describe given experiment data 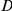 multiplied by the likelihood of prior experimental information about the parameter values themselves. The sampling process is thus based on Bayesian inference of a posterior distribution of parameter sets 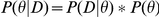, where 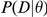 is the likelihood of the data given a parameter set,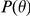 is the prior probability of the parameter set based on experimental prior knowledge on single parameter values and the posterior 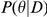
is the probability of a parameter set to describe the given experimental data. The construction of the ensemble with a Markov-Chain Monte Carlo (MCMC) method was done with the Metropolis-Hastings algorithm [54]. The Sloppy cell software environment, used for the analysis, was adapted to process all operators which were in the SBML file describing the model. The modified version is provided in Dataset S1. To speed up convergence, Sloppy Cell takes larger steps along sloppy directions and smaller steps along stiff directions in parameter space; this ‘importance sampling’ is described in [20], [21].

### Experimental data

Measured values of molecular model parameters and their provenance, extracted from the scientific literature, are listed in Table 1. For nine of the 22 parameters reliable standard measurement errors could be found. In addition to the direct measurements on molecular parameters, we employ t_mito_ values from a study by Harrison et al. where the effects of inhibiting creatine kinase and different sizes of electrically paced heart rate jumps in rabbit hearts were investigated [9]. Isolated hearts were perfused with Tyrode's solution containing among others glucose and pyruvate to provide substrates for energy metabolism. In our dataset we include two experimental conditions where hearts were exposed to either (i) iodoacetic acid (IAA) to block glyceraldehyde-3-phosphate dehydrogenase (GAPDH) or (ii) iodacetamide (IA) to inhibit both CK and GAPDH. In order to provide a sufficient amount of reducing equivalents to fuel aerobic respiration despite the inhibition of glycolysis, the buffer also contained pyruvate.

Adenosine was added to the Tyrode buffer to ensure that oxygen supply is non-limiting when oxygen consumption is recorded. The whole heart measurements were corrected for the O_2_ transport time in the coronary vessels based on a model of oxygen transport by convection in blood vessels and diffusion through tissue. The t_mito_ therefore reflects the response time at the level of the mitochondria (cf. [9] and references cited there). The mean response time was also corrected for a small deviation from an ideal step in beat-to-beat ATP hydrolysis measured as an initial overshoot in rate-pressure product [50]. For each condition, steps in heart rate were imposed from 135 to 160, 190 and 220 beats per minute, respectively, using electrical pacing. Note that glycolysis is always inactive when the dynamic response is measured, which corresponds to the absence of glycolysis in the computational model. This approach made it possible to isolate the contribution of the CK system from the contribution of glycolysis, which removes substantial complexity from the model analysis.

A step in ATP hydrolysis from 486.5 to 627.6 µmol*l^−1^ cell water*s^−1^ corresponds to a step in the electrically paced heart rate from 135 to 220 bpm, as was estimated from measurements of myocardial oxygen consumption [18]. From these values, we linearly interpolated hydrolysis rates of 531.4 and 579.5 µmol*l^−1^ cell water*s^−1^ for heart rates 160 and 190 bpm, respectively. To simulate CK inhibition by IA the model parameters for the maximum velocities of both enzyme reactions were set to 2.3% of their original values, corresponding to the CK activity measured for the inhibited hearts. Note that the enzyme activities, the mitochondrial capacities and the whole organ dynamic response times were all measured in the same experimental model by the same laboratory.

### Cost function

Model parameters are fitted to experimental data using a modified Levenberg-Marquardt least-squares procedure in logarithmic parameter space, which is part of the SloppyCell modeling environment. For our model and data we calculate the cost 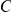 for a given parameter set 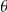 as follows:
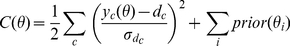
(4)with y_c_ being the model prediction of t_mito_ (Eq. 3) as a function of the parameter value θ and d_c_ the measured value for condition c with standard error 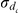. The first term of the cost function takes into account the experimental data on the whole heart level, whereas the second term represents prior experimental information about parameter values found in the literature or measured in conjunction with the modeled experiments. The prior cost, which gives a penalty for a parameter θ_i_ for drifting to far from its measured value θ_i_^*^, is calculated as in [54]:
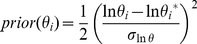
(5)


Note how the prior is used to enter experimentally measured information on parameters measured at the molecular level in the second term of Eq. 4, while the first term contributes measured information on the whole system response. The deviations of the predicted response times from their measured values are penalized relative to their measured standard errors and the deviation of the molecular parameters from measured values are penalized relative to their reported standard errors. Values for molecular parameters reported in the literature are usually given as mean and standard error. However, in the sloppy modeling framework, it is preferable to choose a normal distribution in log space [20], [22], [54]. A Gaussian distribution of logarithmic parameters has been proposed to be biologically plausible [55]. This forms a convenient way to deal with dimensionless positive quantities as parameter values [56].

In order to calculate the σ value for a parameter θ in log space from its reported standard error (considering the span of a 95% confidence region), we set the 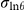 value as follows:
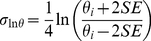
(6)where SE is the absolute standard error of parameter θ_i_. If the standard error is small relative to the mean of the parameter, the shapes of the prior distributions become approximately normal (see Figure 3). Since standard errors for only nine of all 22 system parameters could be found, we chose the default 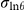 value for the remaining parameters to be at the maximum of all 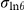 values for parameters with known error. This maximum was the error of the parameter for the binary dissociation constant for creatine from Mi-CK (K_ib,Mi_, and see Table 1). In order to investigate the effect of altered default prior standard deviation on posterior parameter distributions and ensemble predictions, we performed several additional ensemble simulations with lower and higher default 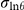 values. Results of these simulations can be found in Text S1. The parameter describing MOM conductance for adenine nucleotides, PS_mom,AdN_, could not be reliably determined by experiments on the organellar level and was therefore not constrained by a prior.

### Determining prediction uncertainty: Ensemble simulations

A first estimate of parameter values was determined by a least-squares fit to the data, using the cost function of equation 4. This initial best parameter estimate resulting from the optimization is used as the starting point for a walk through the parameter space using the Metropolis-Hastings algorithm. Starting the random walk from the optimized set of parameters made the algorithm converge more quickly to the posterior distribution. We use the algorithm's implementation in SloppyCell to sample parameter sets with probability density proportional to 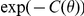. All scripts to reproduce the presented calculations can be found in Dataset S2. To ensure that the members of the ensemble are statistically independent, we ‘prune’ the ensemble by taking only every n^th^ sample, where n is the maximum correlation time of all parameters. The correlation time of a parameter is defined as the time constant of its autocorrelation function. For our model, taking 50000 steps in the random walk is sufficient to obtain more than 600 independent parameter sets. The independent parameter sets in the ensemble provide the final estimate of the parameters, not only characterized by a mean but also by a standard deviation which reflects the spread of the estimation. Calculations were executed in parallel on a ClusterVision parallel machine with 16 nodes of four 3GHz processors with 4GB RAM. For computational performance reasons, we calculated model simulations for parameter estimation and ensemble sampling with an ATP hydrolysis rate averaged over the cardiac cycle rather than the pulsatile pattern shown in Figure 8. This reduced the time needed for calculations tremendously, making it feasible to do the ensemble calculations in several hours.

However, to investigate the damping characteristics of the system, we use a pulsatile forcing function of ATP hydrolysis (see Figure 8A) [18]. To assess the differences in metabolite levels and fluxes caused by replacing the pulsatile function with a time-averaged continuous function, 1000 parameter sets were randomly drawn from all parameter sets tried in the Monte-Carlo random walk, to compare the values of model results between pulsatile and nonpulsatile simulations. The variables most affected by the pulsatile approximation are R_diff,PCr_ and t_mito_. The difference between pulsatile vs. nonpulsatile simulations of all 1000 parameter sets is 7.6±4.3 and 6.8±1.5% (mean±SD), respectively. t_mito_ values from nonpulsatile simulations are always slightly smaller than values from a pulsatile simulation, but their deviation is smaller than the standard error of the experimental t_mito_ data. The difference between pulsatile and non-pulsatile model results for other variables is below 4.5% of their average values in a nonpulsatile setting.

## Supporting Information

Dataset S1 **Patched SloppyCell Python library.** This additional dataset consists of a patched version of the SloppyCell Python library, version 0.8.1, which is required to reproduce all calculations in this manuscript. The package is provided as a zip file. Detailed installation instructions can be found in the zip file.
(ZIP)

Dataset S2 **Model files and Python code.** This zip file contains the model in SBML format and all Python scripts necessary to reproduce the results in this study.
(ZIP)

Text S1 **Ensemble predictions with different default prior standard deviations.** This supplemental text reports the results of our analysis procedure when smaller or larger default prior standard deviations for parameters with unknown standard error are assumed.
(PDF)

Text S2 **Model analysis with additional microcompartment which couples CK to the adenine nucleotide translocator.** In this supplemental text we present the results of the analysis of a computational model which implements substrate channeling between Mi-CK and ANT in a microcompartment, integrated with the data on mitochondrial response times used in this study.
(PDF)
