# Supplementary material for: Analyzing the Functional Properties of the Creatine Kinase System with Multiscale ‘Sloppy’ Modeling
Source: PLoS Comput Biol. 2011 Aug 11;7(8):e1002130. doi: 10.1371/journal.pcbi.1002130 (PMC3166207; doi:10.1371/journal.pcbi.1002130)
Supplement: Dataset S1 — Patched SloppyCell Python library. This additional dataset consists of a patched version of the SloppyCell Python library, version 0.8.1, which is required to reproduce all calculations in this manuscript. The package is provided as a zip file. Detailed installation instructions can be found in the zip file. (ZIP) [file pcbi.1002130.s001.zip › SloppyCell_mod_Hannes_Hettling/Doc/index.html]

SloppyCell


# SloppyCell

SloppyCell is a software environment for simulation and analysis of biomolecular networks developed by the groups of Jim Sethna and Chris Myers at Cornell University.

Examples of models developed in SloppyCell can be found at Jim Sethna's Gene Dynamics page.

## Features

- support for much of the Systems Biology Markup Language (SBML) level 2 version 3
- deterministic and stochastic dynamical simulations
- sensitivity analysis without finite-difference derviatives
- optimization methods to fit parameters to experimental data
- simulation of multiple related networks sharing common parameters
- stochastic Bayesian analysis of parameter space to estimate error bars
  associated with optimal fits

## News

> October 10th, 2007: **New documentation**
> > As part of his thesis, Ryan Gutenkunst has written both user and developer documenation for SloppyCell. They are linked below.

> July 21st, 2007: **SloppyCell 0.81 released**
> > Additions include support for scale factor priors, a new Monte Carlo
> > algorithm that recalculates the sampling matrix each step, and numerous
> > bug fixes.
> >
> > See our download page.

> May 9th, 2007: **SloppyCell 0.8 released!**
> > SloppyCell continues to evolve rapidly.
> >
> > This release sees very signficant speed increases.
> > We've added support for automatically building C versions of all the
> > integration functions. In testing on various networks, this yields a
> > speed up of between a factor of 5 and 20 depending on the application.
> > Additionally, network compilation and sensitivity integration are faster.
> >
> > Finally, algebraic rules are now fully supported, and we have moved
> > completely to using DASKR as our integrator.
> >
> > See our download page.

> January 23rd, 2007: **SloppyCell 0.6 released!**
> > Lots of work over the past few months. The parallel processing code has
> > been completely rewritten. Preliminary support for algebraic rules is also
> > in. (Many thanks to Jordan Atlas for his help.)
> >
> > Grab it from our download page.

## Documentation

- User documentation (pdf)
- Developer documentation (pdf)
- API documentation generated API\_DOC\_GEN\_DATE
- Mailing list

## Miscellaneous

- Sourceforge project page
- Browse the CVS repository
- CVS snapshot (.tar.gz, .zip) generated CVS\_GEN\_DATE
- **Reference:**  
  Ryan N. Gutenkunst, Jordan C. Atlas, Fergal P. Casey, Robert S. Kuczenski, Joshua J. Waterfall, Chris R. Myers, and James P. Sethna.  
  SloppyCell. http://sloppycell.sourceforge.net/ (2007)

$Date: 2007/10/02 01:38:39 $
