# Supplementary material for: Analyzing the Functional Properties of the Creatine Kinase System with Multiscale ‘Sloppy’ Modeling
Source: PLoS Comput Biol. 2011 Aug 11;7(8):e1002130. doi: 10.1371/journal.pcbi.1002130 (PMC3166207; doi:10.1371/journal.pcbi.1002130)
Supplement: Text S1 — Ensemble predictions with different default prior standard deviations. This supplemental text reports the results of our analysis procedure when smaller or larger default prior standard deviations for parameters with unknown standard error are assumed. (PDF) [file pcbi.1002130.s003.pdf]

Analyzing the Functional Properties of the Creatine Kinase System with Multiscale 'Sloppy' Modeling

Supporting Text 1: Ensemble predictions with different default prior standard deviations

Hannes Hettling & Johannes HGM van Beek

Ensemble simulations

For 13 of the 22 model parameters, no reliable standard measurement error could be found in the literature. For those parameters, we assumed the default prior standard deviation corresponding to the highest available measurement error ($\sigma_{ln(\theta)}$ = 0.336; reflecting the reported error of $K_{ib,Mi}$ which is about 30% [1]. In order to test how a different default prior affects posterior parameter distributions and predictions, we performed three additional ensemble predictions with a $\sigma_{ln(\theta)}$ for parameters with unknown errors of (i) 0.107, which is calculated from the mean of all $\sigma_{ln(\theta)}$ for reliably known measurement errors, (ii) 0.637 and (iii) 1.009, which is double and triple the $\sigma_{ln(\theta)}$ for $K_{ib,Mi}$, respectively. Table 1 shows mean parameter values and the prior and posterior standard deviations for all three ensembles. In general, posterior standard deviations increase with a higher prior $\sigma_{ln(\theta)}$. For most parameters, the standard deviation of its posterior distribution in the ensemble (in logspace) is close to the prior. However, some parameters, e.g. $V_{max,Mi,f}$, K_{adp} or K_{pi} only show a slight increase of the standard deviation in the posterior distribution with increased default prior (see Table 1), which indicates that they can be estimated relatively well, since they are more strongly constrained by the data. Remarkably, the posterior standard deviation for $PS_{mom,AdN}$ increases with decreasing default $\sigma_{ln(\theta)}$ for the other parameters. Since a smaller prior value decreases variability of the affected parameters in the sampling process, $PS_{mom,AdN}$, being the only unconstrained parameter, can compensate for the decreased flexibility when fitting the data and therefore its standard deviation in the ensemble may be increased.

Predictions

In order to test the effect of altered default $\sigma_{ln(\theta)}$ values on model predictions of the contributions of PCr to high-energy phosphate transport across the mitochondrial outer membrane and the buffering of ATP synthesis rate and ADP concentrations, we performed ensemble predictions for all three parameter ensembles with the altered default priors. Figure 1 shows the prediction of the relative PCr contribution to high-energy phosphate flux across the mitochondrial outer membrane ($R_{diff,PCr}$) for normal and inhibited CK activity. With a low default prior standard deviation, we predict PCr to contribute not more than 20% to energy transport (Figure 1 A). As expected, the prediction becomes more uncertain when increasing the prior standard deviation. However, at a prior $\sigma_{ln(\theta)}$ of 1.009, although $R_{diff,PCr}$ slightly exceeds 60% during diastole (Figure 1 D), the average predicted PCr contribution still is relatively low at about 25%.

Ensemble predictions of the damping of oscillations in ATP synthesis rate and cytosolic ADP concentrations are shown in Figure 2. The amplitude of the time course of ATP synthesis increases from 117.4 ±10.5 µM*s^{-1} to 431.7 ±19.3 µM*s^{-1} if CK is inhibited, given a low $\sigma_{ln(\theta)}$ of 0.107 (Figure 2A). With the highest prior standard deviation we tested ($\sigma_{ln(\theta)}$ = 1.009), the amplitude of ATP synthesis is higher and the prediction more uncertain (309.9 ±68.1 µM*s^{-1} at active and 720.1 ± 63.6 µM*s^{-1} at inactive CK, see Figure 2G). The effect of higher prior standard deviations on the amplitude of the ADP concentration is smaller than the relative effect on the amplitude of ATP synthesis. At the smallest tested default prior, the prediction of the amplitude in ADP concentration for active and inactive CK conditions is 57.8± 5.7 and 144.5±2.5 µM, respectively (see Figure 2B). The ensemble with the highest default prior standard deviation predicts the amplitude in ADP concentration to be 81.3 ± 24.7 µM (CK active) and 147.2±11.5 µM (CK inactive), which is shown in Figure 2H.

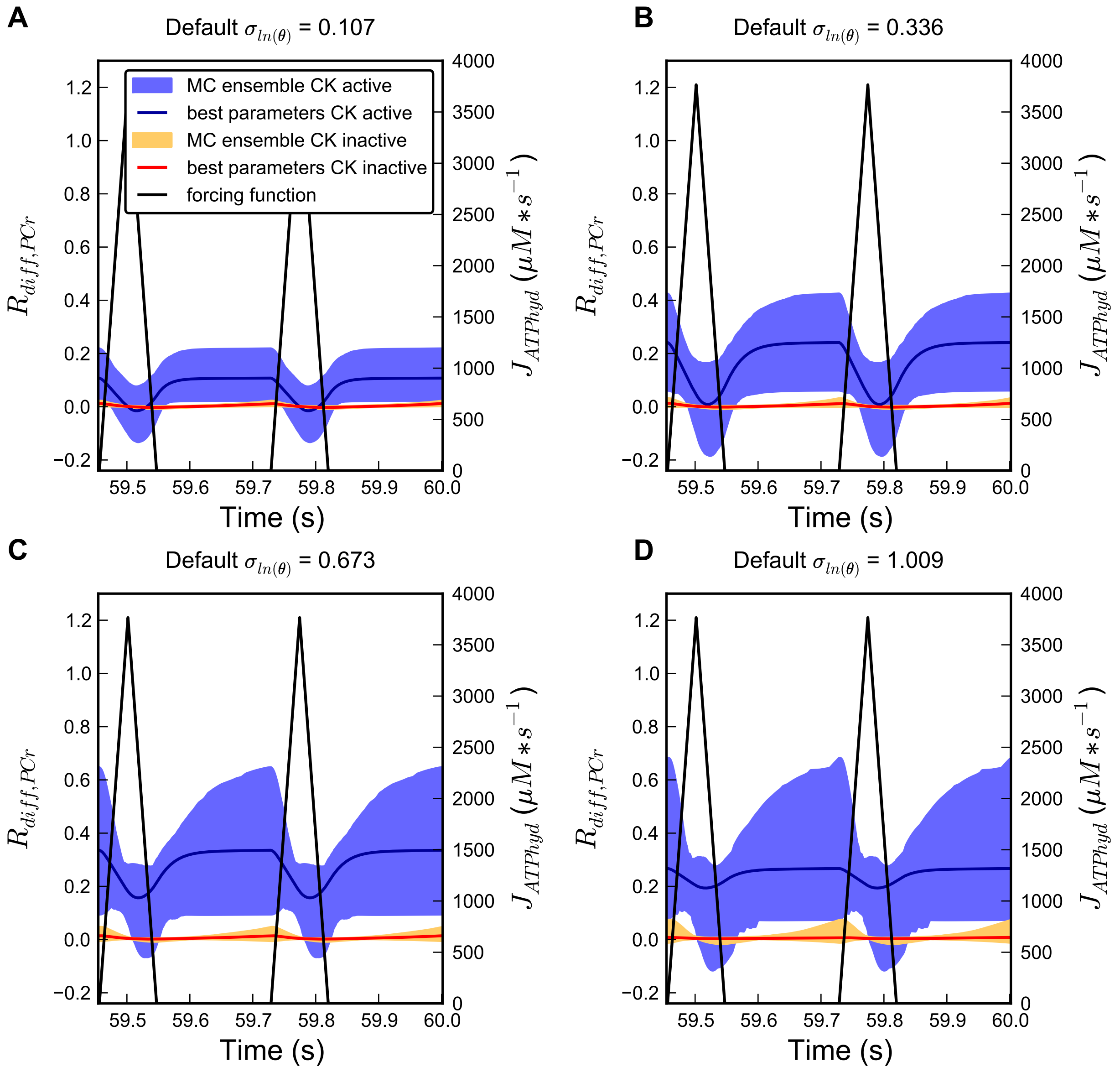

Figure 1. Ensemble predictions of energy transport from mitochondria to cytosol by PCr for different default priors. Prior $\sigma_{ln(\theta)}$ for parameters with unknown standard error was set to (A) 0.107, (B) 0.336, (C) 0.673 and (D) 1.009. Plotted are the relative PCr contribution to high-energy phosphate flux across the mitochondrial outer membrane ($R_{diff,PCr}$) for active and inactive (inhibition by 98%) creatine kinase and the forcing function of pulsatile ATP hydrolysis in the myofibril. Plotted regions represent the 95% confidence interval for all predictions in an ensemble. Please note the different scales for left and right y-axes in each panel.

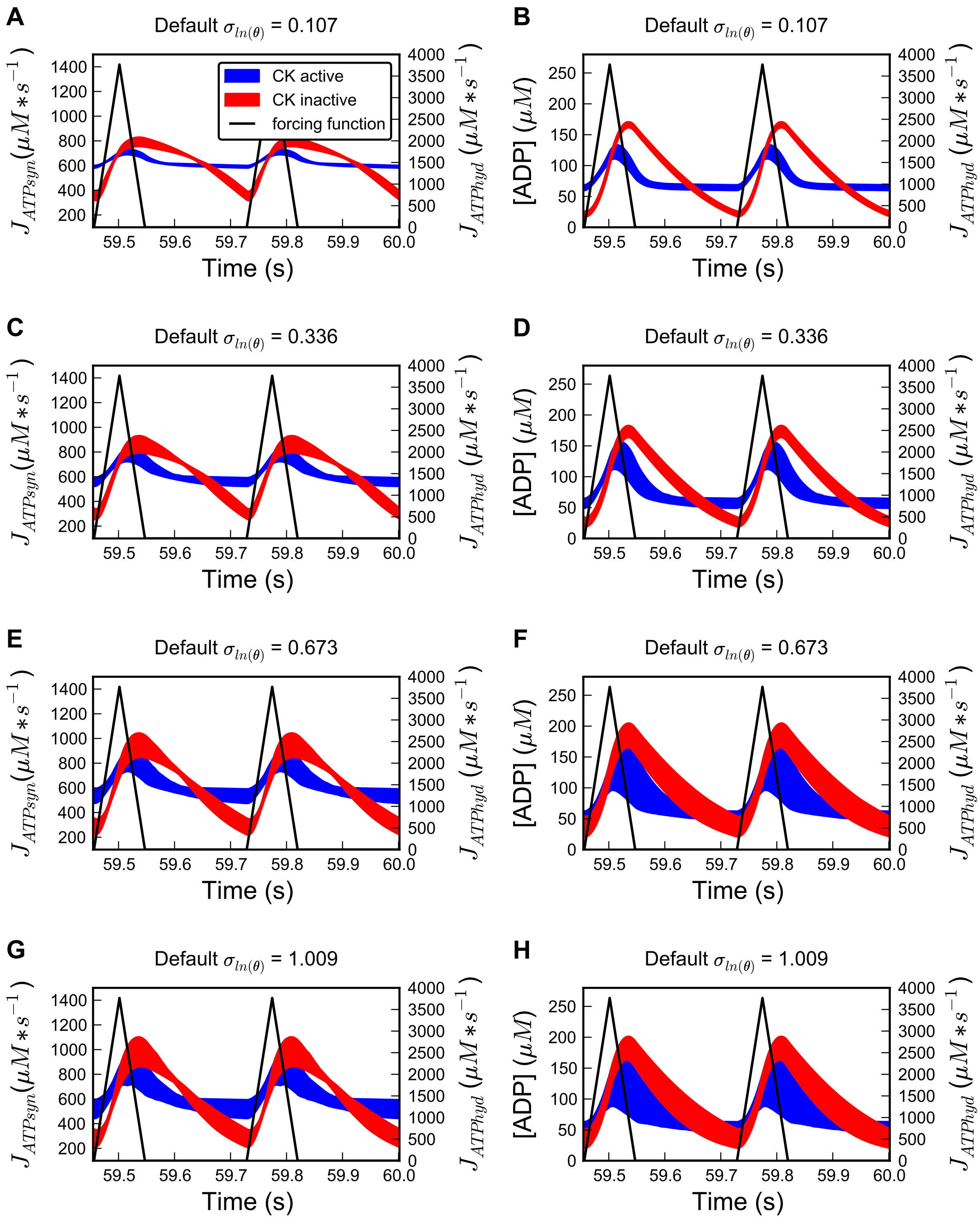

Figure 2. Ensemble predictions of oscillations of ADP levels and oxidative phosphorylation for different default priors.
Predictions of ATP synthesis rate and ADP concentrations from ensembles sampled with prior $\sigma_{\ln(\theta)}$ of (A, B) 0.107, (C, D) 0.336, (E, F) 0.673 and (G, H) 1.009. Blue and red regions show the 95% confidence interval for ensemble predictions with full and inhibited (2% activity) creatine kinase activity, respectively. The forcing function of ATP hydrolysis is plotted in black. Please note the different scales for left and right y-axes in each panel.

Table 1: Priors and posteriors from ensemble simulations with altered default prior standard deviation.

Parameter	Default prior $\sigma_{ln(\theta)}$ = 0.107[a]			Default prior $\sigma_{ln(\theta)}$= 0.336[b]			Default prior $\sigma_{ln(\theta)}$= 0.673[c]			Default prior $\sigma_{ln(\theta)}$= 1.009[d]		
	Ensemble mean±SD	Prior $\sigma_{ln(\theta)}$	Posterior $\sigma_{ln(\theta)}$	Ensemble mean±SD	Prior $\sigma_{ln(\theta)}$	Posterior $\sigma_{ln(\theta)}$	Ensemble mean±SD	Prior $\sigma_{ln(\theta)}$	Posterior $\sigma_{ln(\theta)}$	Ensemble mean±SD	Prior $\sigma_{ln(\theta)}$	Posterior $\sigma_{ln(\theta)}$
$K_{eq,CK}$	152.27±3.91	0.026	0.026	152.32±3.82	0.026	0.025	152.47±3.9	0.026	0.026	152.22±4.13	0.026	0.027
$V_{max,Mi,f}$	886.61±97.52	0.107[a]	0.111	760.05±264.39	0.336[b]	0.333	762.75±393.77	0.673[c]	0.464	668.78±524.62	1.009[d]	0.58
$K_{ia,Mi}$	749.62±61.07	0.081	0.081	754.79±62.61	0.081	0.083	751.8±63.69	0.081	0.084	744.45±60.57	0.081	0.081
$K_{ib,Mi}$	30032.9±10318.4	0.336	0.334	29742±10117	0.336	0.332	30056.72±9295.1	0.336	0.3	31737.3+11647.7	0.336	0.36
$K_{ic,Mi}$	206.48±22.91	0.107[a]	0.11	221.03±79.15	0.336[b]	0.337	254.96±172.29	0.673[c]	0.623	277.93±368.08	1.009[d]	0.948
$K_{id,Mi}$	1604.3±213.01	0.128	0.131	1597.04±190.54	0.128	0.118	1571.7±194.88	0.128	0.124	1619.82±214.37	0.128	0.131
$K_{b,Mi}$	5203.2±286.04	0.058	0.055	5196.36±302.73	0.058	0.058	5213.49±296.48	0.058	0.057	5209.05±308.39	0.058	0.06
$K_{d,Mi}$	502.39±19.91	0.04	0.04	502.19±20.64	0.040	0.041	501.01±19.61	0.04	0.039	498.87±19.25	0.04	0.038
$V_{max,MM,f}$	11391.1±1209.4	0.107[a]	0.106	7769.77±2591.30	0.336[b]	0.308	8937.51±5186.11	0.673[c]	0.581	10207.3±9436.2	1.009[d]	0.715
$K_{ia,MM}$	900.34±97.96	0.107[a]	0.108	1033.59±351.91	0.336[b]	0.336	1353.01±1030.03	0.673[c]	0.669	1559.29±1574.26	1.009[d]	0.892
$K_{ib,MM}$	35045.9±3818.3	0.107[a]	0.109	36772±12695	0.336[b]	0.330	44952.01±34756.14	0.673[c]	0.722	54320.8±69811.6	1.009[d]	0.951
$K_{ic,MM}$	224.17±24.6	0.107[a]	0.11	225.49±78.53	0.336[b]	0.338	256.4±185.15	0.673[c]	0.682	348.16±467.35	1.009[d]	1.008
$K_{id,MM}$	4786.54±481.17	0.107[a]	0.1	4955.05±1692.93	0.336[b]	0.329	5587.71±4428.64	0.673[c]	0.709	6338.79±7203.39	1.009[d]	0.96
$K_{b,MM}$	15868.9±2673.5	0.167	0.166	16869±2940	0.167	0.177	16076.45±2828.23	0.167	0.174	15980.34±2744.0	0.167	0.17
$K_{d,MM}$	1667.44±38.2	0.024	0.023	1670.91±38.38	0.024	0.023	1673.59±37.56	0.024	0.022	1679.14±41.17	0.024	0.025
$V_{max,syn}$	1303.72±55.71	0.103	0.043	1320.53±113.50	0.103	0.085	1399.88±132.62	0.103	0.094	1456.75±151.98	0.103	0.104
K_{adp}	25.8±2.96	0.107[a]	0.114	34.61±7.80	0.336[b]	0.228	35.12±8.91	0.673[c]	0.26	33.2±13.52	1.009[d]	0.415
K_{pi}	799.47±84.7	0.107[a]	0.106	378.88±118.91	0.336[b]	0.296	147.14±58.13	0.673[c]	0.372	107.42±45.63	1.009[d]	0.399
$PS_{mom,AdN}$	200.83±286.48	None	0.971	31.74±16.58	None	0.500	12.49±2.92	None	0.226	11.22±2.32	None	0.194
$PS_{mom,PCr}$	156.65±17.07	0.107[a]	0.109	167.42±57.39	0.336[b]	0.334	184.92±136.09	0.673[c]	0.65	229.31±235.54	1.009[d]	1.037
$PS_{mom,Cr}$	157.18±16.63	0.107[a]	0.105	163.06±59.68	0.336[b]	0.350	194.64±153.8	0.673[c]	0.665	262.71±305.25	1.009[d]	0.963
$PS_{mom,Pi}$	196.77±20.96	0.107[a]	0.105	199.25±68.34	0.336[b]	0.324	243.11±168.61	0.673[c]	0.655	304.62±317.18	1.009[d]	0.99

Shown are mean values and standard deviations, prior and posterior $\sigma_{ln(\theta)}$ values for three parameter ensembles, generated with different prior $\sigma_{ln(\theta)}$values for parameters with unknown standard error.
[a] prior $\sigma_{ln(\theta)}$= 0.107, which is the mean of all $\sigma_{ln(\theta)}$ for the parameters with known standard error.
[b] prior $\sigma_{ln(\theta)}$= 0.336, which is the maximal $\sigma_{ln(\theta)}$for all parameters with known standard error.
[c] prior $\sigma_{ln(\theta)}$= 0.673, which is double the maximal $\sigma_{ln(\theta)}$ for all parameters with known standard error.
[d] prior $\sigma_{ln(\theta)}$= 1.009, which is triple the maximal $\sigma_{ln(\theta)}$ for all parameters with known standard error.
The number of parameter sets in the ensembles after pruning is 463, 658, 459 and 126 for default $\sigma_{ln(\theta)}$ of 0.107, 0.336, 0.673 and 1.009, respectively.

References

1. Jacobus WE, Saks VA (1982) Creatine kinase of heart mitochondria: changes in its kinetic properties induced by coupling to oxidative phosphorylation. Arch Biochem Biophys 219: 167-178.
