# Supplementary material for: Analyzing the Functional Properties of the Creatine Kinase System with Multiscale ‘Sloppy’ Modeling
Source: PLoS Comput Biol. 2011 Aug 11;7(8):e1002130. doi: 10.1371/journal.pcbi.1002130 (PMC3166207; doi:10.1371/journal.pcbi.1002130)
Supplement: Text S2 — Model analysis with additional microcompartment which couples CK to the adenine nucleotide translocator. In this supplemental text we present the results of the analysis of a computational model which implements substrate channeling between Mi-CK and ANT in a microcompartment, integrated with the data on mitochondrial response times used in this study. (PDF) [file pcbi.1002130.s004.pdf]

Analyzing the Functional Properties of the Creatine Kinase System with Multiscale 'Sloppy' Modeling

Supporting Text 2: Model analysis with additional microcompartment which couples CK to the adenine nucleotide translocator

Hannes Hettling & Johannes HGM van Beek

The Mi-CK may be functionally coupled to oxidative phosphorylation via the adenine nucleotide translocator (ANT) [1-3]. Compartmentation of adenine nucleotides in a separate compartment between ANT and Mi-CK cannot completely explain the change in apparent ATP dissociation constants from Mi-CK during activation of oxidative phosphorylation, but phenomenological models and constants for dynamic compartmentation are considered sufficient to describe functional coupling in models of energy fluxes in the cell [3]. We therefore investigate the potential effects of functional coupling on our conclusions by analysis of the experiments with the model of Vendelin et al. [2]. In that model ATP and ADP exchange via the ANT between the mitochondrial matrix and a microcompartment which also gives access to Mi-CK. The extremely small volume of the microcompartment enables a direct substrate-product channeling between ANT and Mi-CK. We simulated the experimental data with the parameters in [2]. For the heart rate steps from 135 to 160, 190 and 220 beats/min t_{mito} was 0.19, 0.19 and 0.2 s, respectively, in the model simulation with CK fully active, to be compared with 3.0, 3.9 and 5.25 s in the experiments. With CK inhibited by 98%, t_{mito} was 14.7, 11.3 and 10.2 s, while these values were 1.8, 2.5 and 3.3 s in the experiments. It is clear that the simulation results of the model of Vendelin et al. differed very much from the experimental values. We then applied our optimization strategy and the t_{mito} became 3.3, 3.4 and 3.5 s for the full CK activity for heart rate steps to 160, 190 and 220 beats/min, and 4.1, 3.8 and 3.7 s with CK inhited by 98%. Results of the initial simulations and model predictions after parameter fitting are shown in Figure 1. The correspondence with the experimental data is still unsatisfactory after parameter adjustment by the optimization procedure.

Next we conducted the optimization with the CK activity values and $V_{max,syn}$, the mitochondrial capacity for ATP synthesis that had been experimentally determined in the rabbit heart preparation. Now the t_{mito} values for full CK activity were simulated well, but although t_{mito} now decreased for steps to 220 beats/min, it still increased for steps to 160 beats/min and did not change at 190 beats/min. Including the microcompartment parameters in the optimization yielded a very similar result. Even if all sixty parameters in the model were allowed to vary during optimization, the behavior of t_{mito} was not much closer to the experimental results. Only if the MMCK activity parameter was allowed to reach a value about 60% higher than the measured value, the behavior of t_{mito} was almost as close to the behavior of the experimental data as our present model described in the main text. However, several optimized kinetic parameters now deviated strongly from the parameter values in [2], for instance the value of parameter u describing the ratio between mitochondrial membrane potential and the protonmotive force across the inner mitochondrial membrane was 60% lower. We conclude that fitting the phenomenological model of Vendelin et al. to the experimental data is very challenging when taking into account measured values of kinetic molecular parameters. Especially the faster mitochondrial response when CK is inhibited is very difficult to predict.

It should be noted that this phenomenological model had not been tested before with experimental data on either the response time of oxidative phosphorylation or inhibition of CK. However, it is clear that in order to describe both the data with which it was originally tested and the present data the phenomenological model [2] needs to be modified. This is the usual cycle of testing models with new experimental data, after which they often need to be modified.

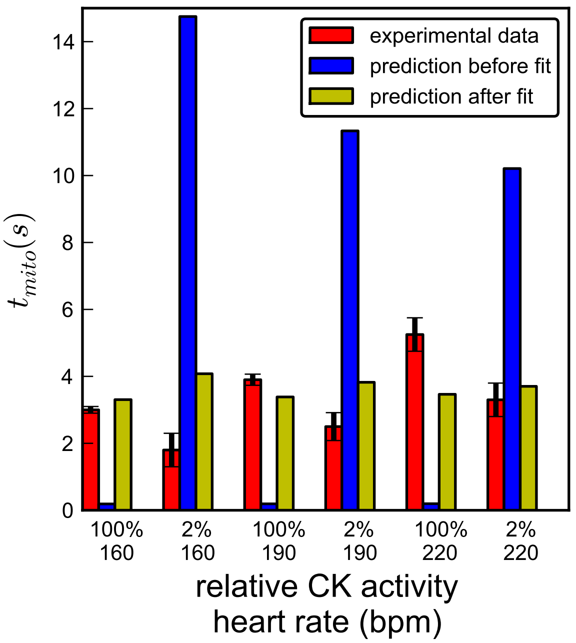

Figure 1: Parameter fit for the model of Vendelin et al. to response times of oxidative phosphorylation measured in isolated rabbit hearts. Model parameters were estimated using a modified Levenberg-Marquardt algorithm. Red bars represent the t_{mito} values from the experiment [4], blue and yellow bars represent the t_{mito} values predicted by the model with the original parameters given in [2] and after parameter adjustment by the fitting procedure, respectively. Data is available for six different conditions: three different amplitudes of heart rate jump (from 135 bpm to 160, 190 and 220 bpm heart rate), each one measured with full wildtype CK activity (100%) or with CK activity inhibited to 2% of wildtype value. Note that in this optimization, all model parameters where allowed to deviate during the fitting procedure.

References

1. Jacobus WE, Saks VA (1982) Creatine kinase of heart mitochondria: changes in its kinetic properties induced by coupling to oxidative phosphorylation. Arch Biochem Biophys 219: 167-178.

2. Vendelin M, Kongas O, Saks V (2000) Regulation of mitochondrial respiration in heart cells analyzed by reaction-diffusion model of energy transfer. Am J Physiol Cell Physiol 278: C747-764.

3. Vendelin M, Lemba M, Saks VA (2004) Analysis of functional coupling: mitochondrial creatine kinase and adenine nucleotide translocase. Biophys J 87: 696-713.

4. Harrison GJ, Wijhe MH van, Groot B de, Dijk FJ, Gustafson LA, et al. (2003) Glycolytic buffering affects cardiac bioenergetic signaling and contractile reserve similar to creatine kinase. Am J Physiol Heart Circ Physiol 285: H883-890.
